# A recurrence prediction model based on serological indicators in children with acute pancreatitis without pancreaticobiliary anatomical abnormalities

**DOI:** 10.3389/fped.2026.1826507

**Published:** 2026-04-28

**Authors:** Xueyi Hu, Tao Zhang, Yuan Cheng, Shiqin Qi, Zhubin Pan

**Affiliations:** 1 Children's Medical Center of Anhui Medical University, Anhui Provincial Children's Hospital, Department of Pediatric General Surgery, Hefei, Anhui, China; 2 The Fifth Clinical Medical College of Anhui Medical University, Hefei, Anhui, China

**Keywords:** acute pancreatitis, blood glucose, children, D-dimer, high density lipoprotein, influencing factors, recurrent acute pancreatitis

## Abstract

**Objective:**

To explore the related influencing factors of recurrent acute pancreatitis in children and provide a reference for clinical early identification of high-risk children with recurrent acute pancreatitis (RAP).

**Methods:**

A retrospective case-control study was designed to collect the clinical data of 101 children who were diagnosed with acute pancreatitis (AP) and hospitalized in Anhui Children's Hospital from July 2020 to December 2025. The children were divided into two groups according to the recurrence of the disease: the acute pancreatitis group (only one hospitalization due to AP during the study period, *n* = 68) and the recurrent acute pancreatitis group (hospitalization due to repeated AP history with an interval of at least one month between any two AP-related hospitalizations, *n* = 33). Demographic data and serological indicators were compared between the two groups. Differential indicators were screened by univariate analysis, and independent related factors of RAP were identified by multivariate binary Logistic regression model. The diagnostic value of independent factors was analyzed by receiver operating characteristic (ROC) curve, and a combined diagnostic model and nomogram were constructed and their efficacy was verified.

**Results:**

Univariate analysis showed that there were statistically significant differences in triglyceride (TG), high density lipoprotein (HDL), blood glucose (BG) and D-dimer levels between the two groups (*P* < 0.05). Multivariate binary Logistic regression analysis indicated that HDL (*OR*=0.037, *95% CI*: 0.003–0.384, *P* = 0.006), BG (*OR*=2.503, *95% CI*: 1.413–4.435, *P* = 0.002) and D-dimer (*OR*=1.525, *95% CI*: 1.199–1.938, *P* < 0.001) were independent related factors for RAP in children. ROC curve analysis showed that the areas under the curve (AUC) of HDL, BG and D-dimer for single diagnosis of RAP were 0.778, 0.830 and 0.825, respectively, and the AUC of the combined diagnosis of the three was 0.943 (*95% CI*: 0.902–0.983, *P* < 0.001). At the maximum Youden index, the sensitivity and specificity of the combined diagnostic model were 0.82 and 0.84, respectively. The calibration curve of the nomogram model constructed based on the above three independent factors had a high coincidence with the diagonal line, suggesting a good predictive efficacy of the model.

**Conclusion:**

HDL is an independent protective factor for RAP in children, while BG and D-dimer are independent risk factors. Clinical combined detection of these three indicators can improve the accuracy of early diagnosis of RAP and provide a basis for precise intervention in high-risk children.

## 1 Introduction

Acute pancreatitis (AP) is one of the common acute abdominal diseases of the digestive system in childhood, with a global incidence showing an annual increase of 5.44% and currently approaching the adult level, and a slightly higher proportion in male children (1). Symptomatic supportive therapy combined with etiological intervention has long been the conventional treatment regimen for pediatric AP, which can effectively relieve acute symptoms, but some children still face the risk of disease recurrence. In recent years, a number of clinical studies have confirmed that the occurrence of recurrent acute pancreatitis (RAP) is closely related to pancreatic structural damage, metabolic disorders and other factors, and children with RAP have a significantly increased risk of progressing to chronic pancreatitis (CP) and diabetes (2). In addition, basic research has proposed that pancreatic tissue in children has important digestive functions and potential immune regulatory effects, and long-term repeated inflammatory damage may affect children's growth and development (3), which has challenged the traditional perception of “only focusing on the acute phase treatment of AP and ignoring the prevention and control of recurrence risk”.

Compared with simple AP, RAP not only brings children distress from recurrent symptoms such as abdominal pain and vomiting, but also increases the risk of long-term complications and the medical burden on families. Therefore, early identification of high-risk individuals with RAP among AP children is of great significance for formulating individualized prevention and control strategies and reducing the recurrence rate. At present, serological and imaging indicators are widely used in the diagnosis and treatment of pediatric AP, including TG, BG, C-reactive protein (CRP), D-dimer, pancreatic ultrasound features, etc (4). Computed tomography (CT) and magnetic resonance imaging (MRI) are not used as routine reexamination and risk assessment items due to radiation exposure or high cost. This study intends to collect clinical data and serological indicators of AP children in our hospital, compare and analyze the differences in indicators between AP and RAP children, screen the independent influencing factors of RAP and verify their predictive value, so as to achieve the goal of early identification of high-risk children with RAP and optimize the clinical diagnosis and treatment of pediatric AP.

## 2 Materials and methods

### 2.1 General information

This was a retrospective study that collected the clinical data of 101 children diagnosed with AP and hospitalized in Anhui Children's Hospital from July 2020 to December 2025. Among all children, there were 56 males and 45 females, with an age range of 1.30–17.10 years and a median age of 8.70 years. The children with AP were divided into two groups according to the presence of recurrence: the AP group (only one AP-related hospitalization during the study period, *n* = 68) and the RAP group (hospitalization due to repeated AP history with an interval of at least one month between any two AP-related hospitalizations, *n* = 33). Informed consent was obtained from all children and their families, and the study was approved by the Ethics Committee of Anhui Children's Hospital.

### 2.2 Inclusion and exclusion criteria

#### 2.2.1 Inclusion criteria

① Age ≤ 18 years; ② Conforming to the diagnostic criteria of pediatric AP or RAP; ③ Complete clinical data, ultrasound or CT examination data, etc.

#### 2.2.2 Exclusion criteria

① Discharged with a diagnosis of chronic pancreatitis; ② Complicated with hepatic and renal insufficiency; ③ Complicated with immune diseases; ④ Complicated with diabetes; ⑤ Anatomical abnormalities of the pancreaticobiliary duct（Patients with anatomical abnormalities of the pancreaticobiliary duct were excluded to avoid confounding bias, as they represent well-defined structural causes unrelated to serological marker-based prediction.) ; ⑥ Incomplete clinical data.

#### 2.2.3 Diagnostic criteria for pediatric AP

Referring to the diagnostic criteria for pediatric AP formulated by the International Study Group of Pediatric Pancreatitis: In Search for a Cure (*INSPPIRE*), the diagnosis of AP requires at least 2 of the following 3 features: ① Abdominal pain symptoms consistent with AP; ② Serum amylase and/or lipase more than 3 times the upper limit of the normal value; ③ Abdominal imaging findings consistent with the imaging characteristics of AP (5).

#### 2.2.4 Diagnostic criteria for pediatric RAP

Two episodes of AP with an interval of at least one month; or if symptoms resolve with complete normalization of pancreatic enzymes, the interval may be less than one month (6).

### 2.3 Data collection

Clinical data of all children during hospitalization were recorded, including general characteristics: gender, age, body mass index (BMI), symptoms at admission, and recurrence status (obtainable from the basic data registered at admission); serological indicators (test results within 24 h of admission): serum amylase, serum lipase, TG, HDL, BG, white blood cell (WBC), albumin, CRP, total bilirubin, alanine aminotransferase (ALT), aspartate aminotransferase (AST), D-dimer. All data were obtained at the first episode of acute pancreatitis and within 24 h of admission.

### 2.4 Statistical methods

IBM SPSS Statistics 31.0 was used for statistical analysis. Measurement data (such as age, HDL) in this study were described as mean ± standard deviation (*x* *±* *s*), and the independent samples t-test was used for comparison between the two groups (for normal distribution and homogeneous variance). The chi-square test was used for comparison of count data (such as gender). First, univariate analysis was performed on the measurement and count data of the two groups, and variables with *P* < 0.05 in univariate analysis were included in the multivariate binary Logistic regression analysis. Finally, *ROC* curve analysis was performed to calculate the *AUC*. A *P* value < 0.05 was considered statistically significant. Based on the independent related factors of pediatric RAP, R language version 4.5.1 was used to draw a nomogram, and the actual and theoretical predicted incidence probabilities of pediatric RAP were compared by calibration curve.

## 3 Results

### 3.1 Baseline levels

A total of 101 children were enrolled, including 68 in the AP group (40 males and 28 females) with a mean age of 9.03 ± 3.70 years, and the levels of D-dimer, HDL, TG and BG within 24 h of admission were 2.16 ± 2.25 mg/L, 1.38 ± 0.48 mmol/L, 1.07 ± 0.44 mmol/L and 5.01 ± 0.97 mmol/L, respectively.

There were 33 children in the RAP group (16 males and 17 females) with a mean age of 8.00 ± 3.62 years, and the levels of D-dimer, HDL, TG and BG within 24 h of admission were 6.90 ± 5.03 mg/L, 0.92 ± 0.25 mmol/L, 1.36 ± 0.59 mmol/L and 6.89 ± 1.94 mmol/L, respectively. There were statistically significant differences in the levels of D-dimer, HDL, TG and BG between the two groups (*P* < 0.05) See Tables 1, 2.

**Table 1:** General clinical data.

Variable	AP group (*n* = 68)	RAP group (*n* = 33)	Statistic	*P* value
Age (years)	9.03 ± 3.70	8.00 ± 3.62	1.324	0.988
Male	40 (58.8%)	16 (48.5%)	0.975	0.328
Female	28 (41.2%)	17 (51.5%)		
BMI	19.56 ± 2.97	20.23 ± 3.46	0.987	0.326
Abdominal pain	54 (79.4%)	28 (84.8%)	0.676	0.501
Abdominal distension	41 (60.3%)	22 (66.7%)	0.608	0.545
Vomiting	51 (75.0%)	23 (69.7%)	0.558	0.578

**Table 2:** Comparison of hematological data between children with AP and RAP.

Indicator	AP group	RAP group	Statistic	*P* value
Serum amylase (U/L)	339.94 ± 261.13	494.12 ± 657.30	−1.691	0.059
Serum lipase (U/L)	287.68 ± 256.14	373.33 ± 343.91	−1.405	0.053
WBC (×10^9/mL)	12.76 ± 6.14	12.49 ± 7.05	0.463	0.199
D-dimer (mg/L)	2.16 ± 2.25	6.90 ± 5.03	−5.163	<0.001
CRP (mg/L)	25.88 ± 38.38	25.04 ± 44.89	0.097	0.408
Triglyceride (mmol/L)	1.07 ± 0.44	1.36 ± 0.59	−2.476	0.016
HDL (mmol/L)	1.38 ± 0.48	0.92 ± 0.25	6.261	0.001
AST (U/L)	87.55 ± 143.64	100.76 ± 152.94	−0.424	0.497
ALT (U/L)	99.99 ± 178.52	84.45 ± 131.30	0.445	0.464
Albumin (g/L)	44.30 ± 4.53	44.23 ± 4.01	0.080	0.465
Total bilirubin (µmol/L)	19.89 ± 26.57	20.86 ± 21.79	0.982	0.183
Blood glucose (mmol/L)	5.01 ± 0.97	6.89 ± 1.94	−5.265	<0.001

*P* < 0.05 indicates a statistically significant difference.

#### 3.1.1 Recurrence and etiology in the RAP group

Among the 33 children in the RAP group, 21 patients had 2 episodes of acute pancreatitis, and 12 patients had 3 or more episodes. The etiological distribution was as follows: idiopathic pancreatitis in 23 cases, hypertriglyceridemia-induced pancreatitis in 7 cases, and infection-related or systemic factors in 3 cases. All patients had no anatomical abnormalities of the pancreaticobiliary duct.

### 3.2 Multivariate logistic regression analysis of RAP

Multivariate Logistic regression analysis was performed on the above indicators with statistical significance, with AP recurrence as the dependent variable (assignment: recurrence group=1, non-recurrence group=0). The results showed that HDL (*OR*=0.037, *95% CI*: 0.003–0.384, *P* = 0.006), BG (*OR*=2.503, *95% CI*: 1.413–4.435, *P* = 0.002) and D-dimer (*OR*=1.525, *95% CI*: 1.199–1.938, *P* < 0.001) were three independent influencing factors. Among them, HDL was an independent protective factor for the recurrence of AP in children (*P* < 0.05), and BG and D-dimer were independent risk factors (*P* < 0.05) See Table 3.

**Table 3:** Logistic regression analysis.

Factor	B	SE	OR	*P* value	95% CI
HDL	−3.307	1.199	0.037	0.006	0.003–0.384
Blood glucose	0.917	0.292	2.503	0.002	1.413–4.435
D-dimer	0.422	0.123	1.525	<0.001	1.199–1.938

*P* < 0.05 indicates a statistically significant difference.

### 3.3 ROC curve analysis

**Figure 1:**
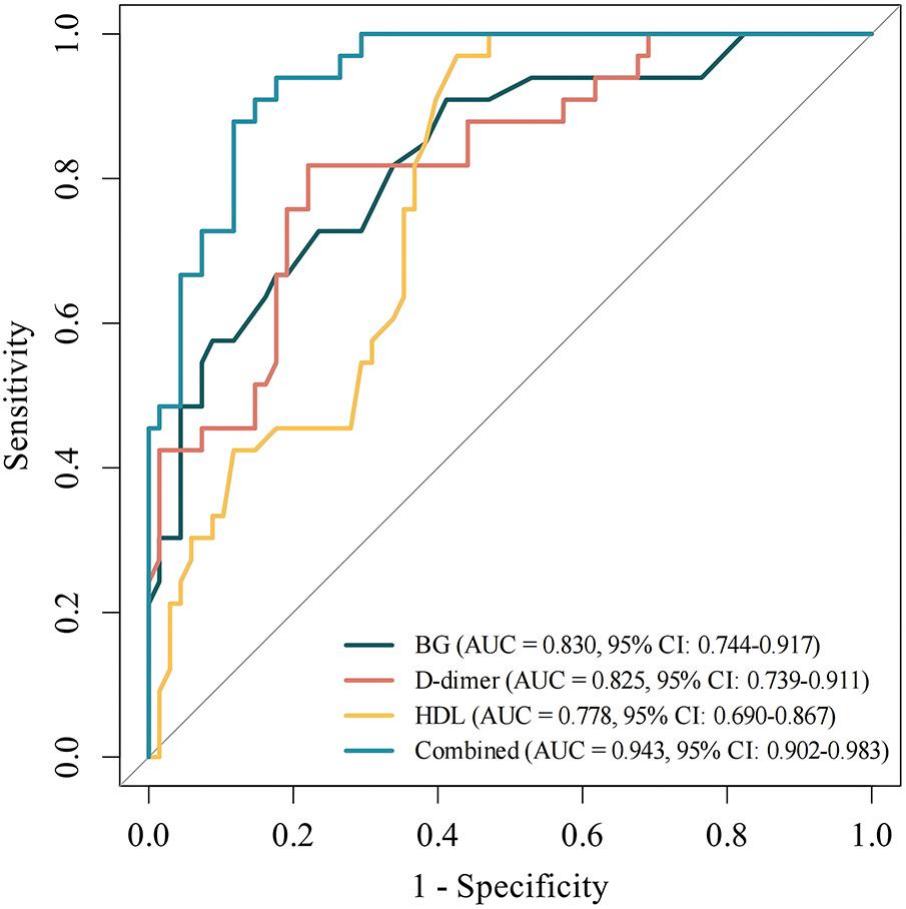
ROC curve shows the predictive accuracy of serum biomarkers (D-dimer, BG, HDL within 24 h of admission) for the possible recurrence of acute pancreatitis in children.

ROC curve analysis of RAP prediction parameters showed that the AUC of HDL, BG and D-dimer were 0.778, 0.830 and 0.825, respectively (*P* < 0.05). The AUC of the combined prediction parameters was 0.943 (*P* < 0.05), with a sensitivity of 0.82 and a specificity of 0.84 for combined prediction See Figure 1.

### 3.4 Nomogram construction

Based on the results of multivariate binary Logistic regression analysis, this study finally identified three independent influencing factors: HDL, BG and D-dimer. A nomogram model was constructed by combining the above three independent influencing factors.

The application method of the nomogram is as follows:

First, the corresponding scores of BG, HDL, and D-dimer levels were determined according to the scales in the nomogram.

Second, the total score was calculated by summing up the scores of the three indicators.

**Figure 2:**
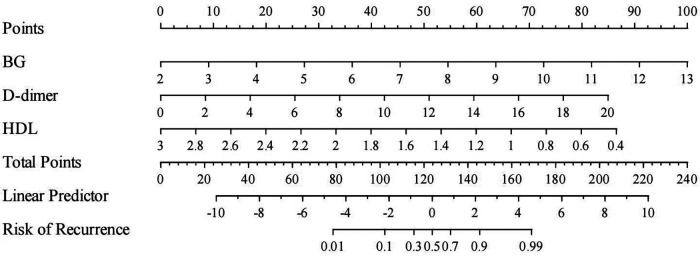
Nomogram for predicting the risk of recurrent acute pancreatitis in children. Each variable was assigned a score, and the total score corresponded to the probability of recurrence.

Finally, a vertical line was drawn from the total score to the predicted probabilit*y* axis to obtain the individual risk probability of RAP in children with AP (Figure 2).

### 3.5 Calibration curve results

**Figure 3:**
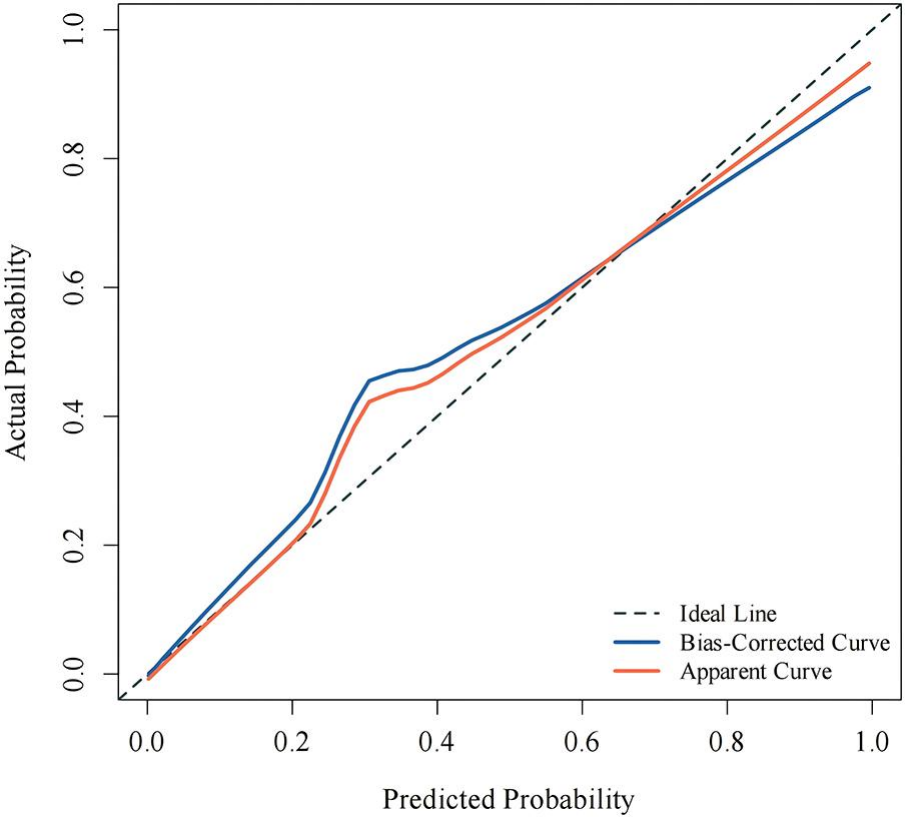
Calibration curve of the recurrence prediction model. The diagonal line represents an ideal prediction; the solid line shows the actual predictive performance of the model.

In this study, the Apparent and Bias-corrected curves were close to the reference line, indicating that the predicted probability was highly consistent with the actual incidence probability, and the model had high predictive value and could be applied in practice (Figure 3).

### 3.6 Decision curve analysis (DCA) results

**Figure 4:**
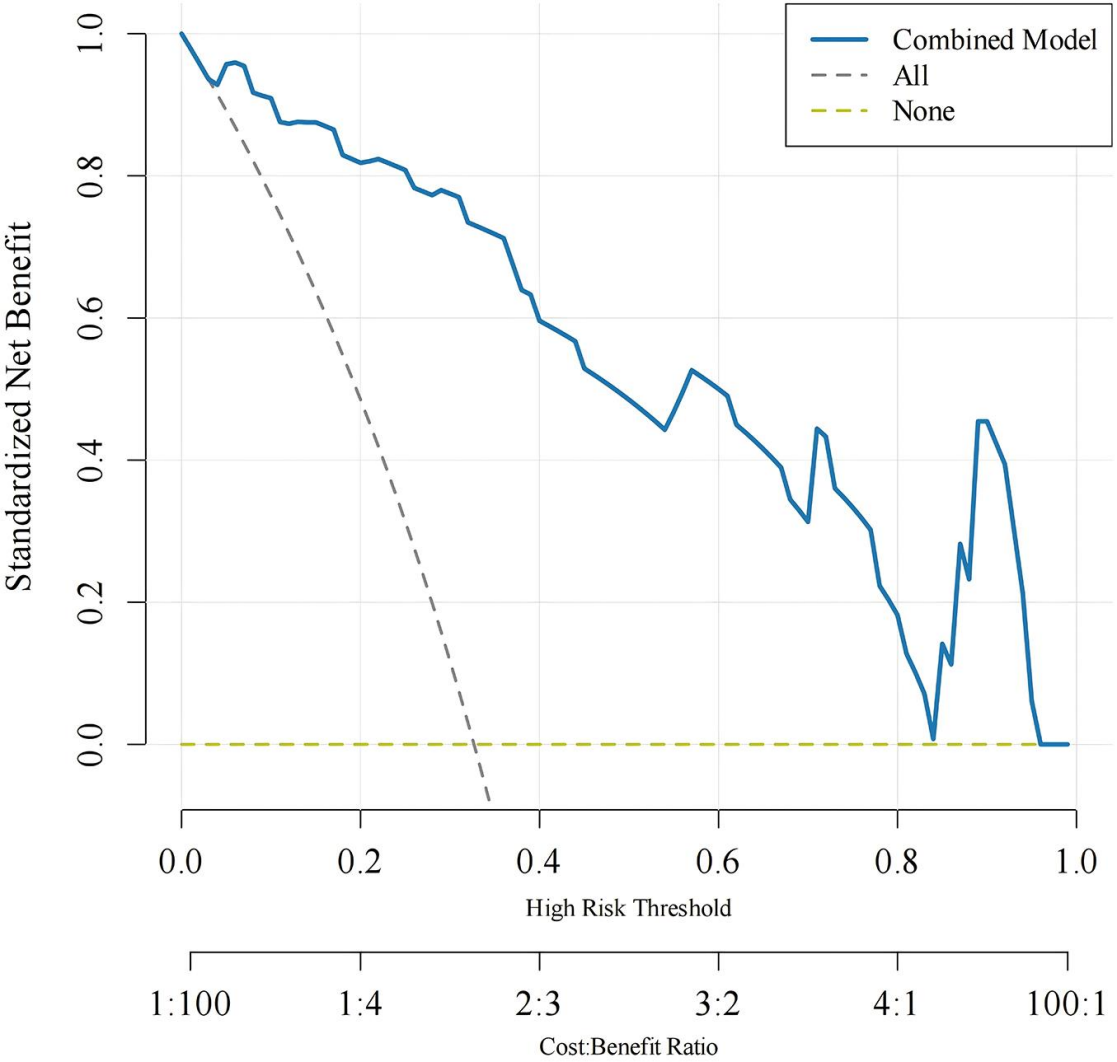
Decision curve analysis (DCA) of the prediction model. The net benefit was evaluated across different threshold probabilities to assess the clinical utility of the model.

The prediction model constructed in this study showed that the blue curve had a higher net benefit than the diagonal line within a wide range of thresholds, which also confirmed the high predictive value of the model (Figure 4).

## 4 Discussion

Pediatric AP is a relatively rare digestive system disease in children, characterized by sudden persistent upper abdominal pain accompanied by gastrointestinal symptoms. RAP is a special type of AP with an incidence of approximately 13.1% in Asian children, which can progress to CP. In addition, the incidence of pediatric AP has been increasing in recent years, with a mortality rate of up to 11% (5, 7). At present, there are still great difficulties in the early identification of children with RAP in clinical practice, making it impossible to implement timely intervention to improve the prognosis of children. Existing studies on the potential risks of RAP mostly focus on pancreaticobiliary structural abnormalities and hematological parameters (8), and the research objects are mainly adults. There is still a lack of research on the combined prediction of serological indicators for pediatric RAP, and the research conclusions are difficult to be directly applied to clinical practice in children. To solve the above problems, this study adopted a retrospective case-control design to collect clinical data of 101 children with AP, who were divided into the AP group (*n* = 68) and the RAP group (*n* = 33) according to disease recurrence. Relevant indicators were compared between the two groups and statistical analysis was performed to explore effective indicators for early prediction of RAP. The results showed that HDL, BG and D-dimer were independent related factors for pediatric RAP, and the *AUC* of their combined diagnosis reached 0.943 with good diagnostic efficacy. The nomogram constructed based on the above indicators can be used for the early prediction of high-risk children with RAP, providing a solid basis for clinical intervention.

Compared with pancreaticobiliary structural abnormalities and imaging parameters, hematological indicators have higher research value and clinical practicability in the risk prediction of RAP. Relevant literature has confirmed that hematological parameters have more objectivity, convenient detection, and advantages in sensitivity and specificity compared with imaging parameters, which can effectively avoid subjective judgment bias and long detection time in imaging examinations, and are more suitable for early screening and risk prediction of RAP (9). Current studies have shown that potential hematological indicators affecting RAP include serum amylase, serum lipase, total cholesterol, BG, HDL, low density lipoprotein (LDL), TG, etc (10). In this study, we focused on the advantageous detection direction of hematological indicators, and found that there were significant differences in HDL, BG, TG and D-dimer between the AP group and the RAP group in the hematological indicators within 24 h of admission (all *P* < 0.05). Among them, elevated BG and D-dimer within 24 h of admission were risk factors for the recurrence of pediatric AP, and elevated HDL within 24 h of admission was a protective factor, which may indicate that BG, HDL and D-dimer are three potential predictive factors.

HDL is a complex lipoprotein composed of lipids, proteins and their carried regulatory factors (11), mainly synthesized in the liver, and has the functions of mediating cholesterol transport to the liver, anti-inflammation, anti-oxidation and protecting endothelial cells. In the results of this study, the HDL level in the AP group was 1.38 ± 0.48 mmol/L and that in the RAP group was 0.92 ± 0.25 mmol/L, with a statistically significant difference between the two groups (*OR*=0.037, *95% CI*: 0.003–0.384, *P* = 0.006), indicating that HDL is an independent protective factor for the recurrence of AP in children. This is because children with RAP usually experience multiple episodes of inflammation during the onset, and the anti-inflammatory and antioxidant effects of HDL can reduce the occurrence of inflammation in the body (12, 13), thereby reducing the risk of progressing to RAP. Similarly, Li et al. found that the incidence of metabolic syndrome (MetS) was higher in patients with RAP, and the proportion of HDL levels lower than 1 mmol/L increased significantly (14). However, some studies have shown that HDL may decrease temporarily in the acute inflammatory phase of AP, suggesting that the transient decrease of HDL in the acute inflammatory phase of AP may not be the direct cause of AP occurrence, and its causal relationship with pediatric RAP needs to be further studied and clarified (15). In addition, HDL level is closely related to TG level, and the decrease of HDL level may lead to hypertriglyceridemia. Wu et al. found that TG may be a known risk factor for RAP (16).

Similarly, in this study, the TG level in the AP group was 1.07 ± 0.44 mmol/L and that in the RAP group was 1.36 ± 0.59 mmol/L, with a statistically significant difference between the two groups.

Current studies have shown that hyperglycemia is closely related to pancreatic diseases (17), and BG-related diseases show a younger trend worldwide. In this study, the BG level in the AP group was 5.01 ± 0.97 mmol/L and that in the RAP group was 6.89 ± 1.94 mmol/L, with a statistically significant difference between the two groups (*OR*=2.503, *95% CI*: 1.413–4.435, *P* = 0.002), indicating that BG is an independent risk factor for the recurrence of AP in children. Hyperglycemia may promote the recurrence of pancreatitis through a variety of pathways: on the one hand, the hyperglycemic environment can induce oxidative stress and the release of inflammatory factors, aggravating pancreatic microcirculation disorders (18); on the other hand, insulin resistance may activate trypsinogen and aggravate the process of pancreatic autodigestion, both of which may lead to the transformation of AP patients to RAP (19). Similarly, Shen et al. found in a study on adult RAP that hyperglycemia was also an independent risk factor for recurrent AP attacks (20), which was consistent with the results of our study in children. In addition, a retrospective study found that there were significant changes in the BG level of children with AP, indicating that BG level may play a certain role in the disease process, and metabolic diseases such as obesity and diabetes are also important risk factors for the occurrence of pediatric RAP (21).

Abnormal coagulation function is an important inherent pathophysiological change of AP, and the lesion can progress from local intravascular microthrombosis to severe disseminated intravascular coagulation (DIC), and the degree of coagulation disorder is closely related to the severity of the disease (22). As a specific marker of the activation of the coagulation-fibrinolysis system, D-dimer can directly reflect the activation of the coagulation system and the state of secondary fibrinolysis hyperactivity in the body, and has been confirmed to have the value of early disease assessment and prognosis prediction in adult AP (23). Elevated D-dimer levels in AP patients indicate that the body is in a hypercoagulable state and microvascular coagulation dysfunction, which is prone to induce the formation of microthrombus in pancreatic microcirculation, further aggravate pancreatic tissue damage, increase the risk of local complications, and eventually progress to multiple organ failure and overt DIC (24). The results of this study showed that the D-dimer level in the AP group was 2.16 ± 2.25 mg/L and that in the RAP group was 6.90 ± 5.03 mg/L, with a statistically significant difference between the two groups; multivariate Logistic regression analysis suggested that D-dimer was an independent risk factor for the recurrence of pediatric AP *(OR*=1.525, *95% CI*: 1.199–1.938, *P* < 0.001). Existing studies have confirmed that elevated D-dimer levels are closely related to the severity and poor prognosis of AP. As a marker of the activation of the coagulation-fibrinolysis system, it can indirectly reflect the degree of pancreatic microcirculation disorder and tissue damage, providing an important reference for recurrence risk assessment (23). In adult AP studies, D-dimer has been confirmed to be an independent risk factor for predicting disease progression and recurrence, especially in patients with hyperlipidemic AP, whose elevated levels are significantly associated with an increased risk of recurrence (25). Studies on pediatric AP have also shown that D-dimer levels at admission can effectively assess the severity of the disease and the risk of complications, suggesting that it also has important prognostic value in the pediatric population (26). The above results indicate that elevated D-dimer levels are significantly associated with an increased risk of recurrence of pediatric AP, and can be used as an early serological indicator for predicting the recurrence of pediatric AP.

This study also has some limitations. In this study, patients with pancreaticobiliary anatomical abnormalities were excluded to avoid confounding bias, allowing the model to focus on serological prediction for idiopathic and metabolic RAP. The limitations include a small sample size (*n* = 101) and a single-center retrospective design, which may lead to selection bias; the failure to include genetic, imaging or long-term follow-up data makes it difficult to comprehensively evaluate the multi-factor mechanism of RAP; and the failure to analyze potential influencing factors such as genetic factors and intestinal microecology may lead to the omission of some risk factors for pediatric RAP. In the future, large-sample, multi-center prospective studies are still needed to explore the influencing factors of recurrence in children with AP.

In conclusion, it is necessary to early identify children with AP at risk of recurrence. In our study, BG, HDL and D-dimer have high predictive value for the occurrence of RAP in children with AP. For children with AP, close attention should be paid to BG, HDL and D-dimer within 24 h of admission, and early intervention should be implemented for high-risk children to reduce the risk of RAP and improve the poor prognosis of children with AP.

## Data Availability

The original contributions presented in the study are included in the article/supplementary material, further inquiries can be directed to the corresponding author.

## References

[1] Hu Q Hu Y Tan C Yang Y Su H Huang Z Acute pancreatitis: mechanisms and therapeutic approaches. Signal Transduct Target Ther. (2026) 11:15. 10.1038/s41392-025-02394-6 41530118 PMC12800257

[2] Ahmed F Abu-El-Haija M. Acute pancreatitis in children: it’s not just a simple attack. Gastroenterology. (2025) 169:572–84. 10.1053/j.gastro.2025.04.001 40228704 PMC12353169

[3] Dimou N Peruchet-Noray L Mariosa D Lu Y Gentiluomo M Campa D A Mendelian randomization study of lifestyle factors and glycemic traits and risk of pancreatic cancer. Pancreatology. (2022) 22(Suppl 1):e45. 10.1016/j.pan.2022.06.117

[4] Cho IR Do MY Han SY Jang S Ill. Comparison of interleukin-6, C-reactive protein, procalcitonin, and the computed tomography severity index for early prediction of severity of acute pancreatitis. Gut Liver. (2023) 17(4):629–37. 10.5009/gnl220356 36789576 PMC10352050

[5] Uc A Perito ER Pohl JF Shah U Abu-El-Haija M Barth B International Study Group of Pediatric Pancreatitis: in search for a CuRE cohort study: design and rationale for INSPPIRE 2 from the consortium for the study of chronic pancreatitis, diabetes, and pancreatic cancer. Pancreas. (2018) 47(10):1222–8. 10.1097/MPA.0000000000001172 30325861 PMC6195325

[6] Kumar S Ooi CY Werlin S Abu-El-Haija M Barth B Bellin MD Risk factors associated with pediatric acute recurrent and chronic pancreatitis: lessons from INSPPIRE. JAMA Pediatr. (2016) 170(6):562–9. 10.1001/jamapediatrics.2015.4955 27064572 PMC5317277

[7] Abu-El-Haija M Sliva CM Uc A Dillman JR Perito ER Pohl JF Progression from acute to chronic pancreatitis in children: a systematic review and meta-analysis. Clin Exp Pediatr. (2026) 129(1):1–12. 10.1186/s13052-025-01879-x PMC12877302 41381081

[8] Dillman JR Abu-El-Haija M Uc A Perito ER Pohl JF Barth B Current state of imaging of pediatric pancreatitis: aJR expert panel narrative review. AJR Am J Roentgenol. (2022) 219(3):345–54. 10.2214/AJR.21.27654 PMC8441844 33728974

[9] Singh P Sharma P Kumar S Chui JN Ziaziaris WA Nahm CB Radiological and biochemical parameters in assessing acute pancreatitis severity: a comprehensive review. Pancreas. (2025) 54(7):989–1002. 10.1097/MPA.0000000000002345

[10] Perito ER Uc A Pohl JF Abu-El-Haija M Barth B Bellin MD Demographics and risk factors for pediatric recurrent acute pancreatitis. Curr Gastroenterol Rep. (2021) 23(12):1–8. 10.1007/s11894-021-00876-x33389241

[11] Kontush A Chapman MJ. High-density lipoprotein revisited: biological functions and clinical relevance. Cardiovasc Res. (2025) 121(12):1897–914. 10.1093/cvr/cvad245

[12] Albai O Roman D Frandes M. Hypertriglyceridemia, an important and independent risk factor for acute pancreatitis in patients with type 2 diabetes mellitus. Ther Clin Risk Manag. (2017) 13:515–22. 10.2147/TCRM.S134996 28450786 PMC5399973

[13] Chen Y Huang S Luo B Jiang J Zou K Zhong X Prediction and evaluation of a nomogram model for recurrent acute pancreatitis. Eur J Gastroenterol Hepatol. (2024) 36(5):554–62. 10.1097/MEG.0000000000002732 38407842

[14] Li XQ Liu H Xiao CT Wang Y Zhang L Zhao Y The impact of metabolic syndrome on the recurrence of hyperlipidemic acute pancreatitis patients. J Clin Hepatol Biliary Dis. (2020) 36(11):2515–20. 10.3969/j.issn.1001-5256.2020.11.031

[15] Siebel AL Heywood SE Kingwell BA. HDL And glucose metabolism: current evidence and therapeutic potential. Front Pharmacol. (2015) 6:258. 10.3389/fphar.2015.00258 26582989 PMC4628107

[16] Wu BU Batech M Dong EY Smith J Johnson A Lee S Influence of ambulatory triglyceride levels on risk of recurrence in patients with hypertriglyceridemic pancreatitis. Dig Dis Sci. (2019) 64(3):890–7. 10.1007/s10620-018-0226-5 30094622

[17] Wang Y Otaki S Kawabata Y Nishi T Hayashi H Iwahashi T Hyperglycemia in acute pancreatitis: pathophysiological mechanisms and clinical implications. Pancreas. (2024) 53(8):921–8. 10.1097/MPA.0000000000002089

[18] Petrov MS Yadav D. Global epidemiology and holistic prevention of pancreatitis. Nat Rev Gastroenterol Hepatol. (2019) 16(3):175–84. 10.1038/s41575-018-0054-7 30482911 PMC6597260

[19] Talukdar R Sareen A Zhu H Patel N Gupta S Kumar A Release of cathepsin B in cytosol causes cell death in acute pancreatitis. Gastroenterology. (2016) 151(4):747–58. 10.1053/j.gastro.2016.06.04 27519471 PMC5037034

[20] Shen NS Qiao M. Analysis of the correlation between metabolic syndrome and recurrent acute pancreatitis. Chin J Dig. (2024) 44(1):38–43. 10.3760/cma.j.cn311367-20230207-00058

[21] Abu-El-Haija M Lowe ME Uc A Dillman JR Perito ER Pohl JF The role of pancreatitis risk genes in endocrine insufficiency development after acute pancreatitis in children. Clin Gastroenterol Hepatol. (2024) 22(6):1242–50. 10.1016/j.cgh.2023.10.03 PMC11424246 38871151

[22] Levi M van der Poll T. Disseminated intravascular coagulation: the past, present, and future considerations. Semin Thromb Hemost. (2025) 51(1):15–24. 10.1055/s-0044-1778934 36100234

[23] Mannucci PM. How we manage a high D-dimer. Haematologica. (2024) 109(4):1035–42. 10.3324/haematol.2023.283966 37881856 PMC10985443

[24] Juneja SK Tandon P Kaur G. Evaluation of the effect of increasing maternal age on maternal and neonatal outcomes in pregnancies at advanced maternal age. Int J Appl Basic Med Res. (2023) 13(4):239–42. 10.4103/ijabmr.ijabmr_483_23 PMC9886143 36726656

[25] Chen J Dig D Shi H Liu Y Wang H Zhang Y D-dimer as an independent predictor of recurrence in hypertriglyceridemic acute pancreatitis. Dig Dis Sci. (2023) 68(7):2678–85. 10.1007/s10620-023-02189-x

[26] Li X Wang Y Zhang L Liu H Chen J Hu X Clinical characteristics and prognosis of acute pancreatitis in children. Front Pediatr. (2025) 13:1324567. 10.3389/fped.2025.1324567

